# Pyruvate dehydrogenase expression is negatively associated with cell stemness and worse clinical outcome in prostate cancers

**DOI:** 10.18632/oncotarget.14527

**Published:** 2017-01-05

**Authors:** Yali Zhong, Xiaoli Li, Yasai Ji, Xiaoran Li, Yaqing Li, Dandan Yu, Yuan Yuan, Jian Liu, Huixiang Li, Mingzhi Zhang, Zhenyu Ji, Dandan Fan, Jianguo Wen, Mariusz Adam Goscinski, Long Yuan, Bin Hao, Jahn M Nesland, Zhenhe Suo

**Affiliations:** ^1^ Department of Oncology, The First Affiliated Hospital of Zhengzhou University, Zhengzhou, Henan Province, China; ^2^ Department of Gastroenterology, The Second Affiliated Hospital of Zhengzhou University, Zhengzhou, Henan Province, China; ^3^ Department of Pathology, The Norwegian Radium Hospital, Oslo University Hospital, University of Oslo, Montebello, Oslo, Norway; ^4^ Department of Pathology, Institute for Clinical Medicine, Faculty of Medicine, University of Oslo, Oslo, Norway; ^5^ Department of Pathology, Capital Medical University, Beijing, China; ^6^ Institute of Health Quarantine, Chinese Academy of Inspection and Quarantine, Beijing, China; ^7^ Department of Pathology, The First Affiliated Hospital of Zhengzhou University, Zhengzhou, Henan Province, China; ^8^ Henan Academy of Medical and Pharmaceutical Sciences, Zhengzhou University, Zhengzhou; ^9^ Institute of Clinical Medicine, The First Affiliated Hospital of Zhengzhou University, Zhengzhou University, Henan, China; ^10^ Department of Surgery, The Norwegian Radium Hospital, Oslo University Hospital, Oslo, Norway; ^11^ Department of Surgery, The Affiliated Cancer Hospital of Zhengzhou University, Zhengzhou, Henan, China; ^12^ Department of Urology, The Second Affiliated Hospital of Zhengzhou University, Zhengzhou, Henan, China

**Keywords:** PDHA1, glycolysis, stemness, prostate cancer

## Abstract

Cells generate adenosine-5′-triphosphate (ATP), the major currency for energy-consuming reactions, through mitochondrial oxidative phosphorylation (OXPHOS) and glycolysis. One of the remarkable features of cancer cells is aerobic glycolysis, also known as the “Warburg Effect”, in which cancer cells rely preferentially on glycolysis instead of mitochondrial OXPHOS as the main energy source even in the presence of high oxygen tension. One of the main players in controlling OXPHOS is the mitochondrial gatekeeperpyruvate dehydrogenase complex (PDHc) and its major subunit is E1α (PDHA1). To further analyze the function of *PDHA1* in cancer cells, it was knock out (KO) in the human prostate cancer cell line LnCap and a stable KO cell line was established. We demonstrated that *PDHA1* gene KO significantly decreased mitochondrial OXPHOS and promoted anaerobic glycolysis, accompanied with higher stemness phenotype including resistance to chemotherapy, enhanced migration ability and increased expression of cancer stem cell markers. We also examined PDHA1 protein expression in prostate cancer tissues by immunohistochemistry and observed that reduced PDHA1 protein expression in clinical prostate carcinomas was significantly correlated with poor prognosis. Collectively, our results show that negative *PDHA1* gene expressionis associated with significantly higher cell stemness in prostate cancer cells and reduced protein expression of this gene is associated with shorter clinical outcome in prostate cancers.

## INTRODUCTION

Prostate cancer is the second most frequently diagnosed cancer and the fifth leading cause of cancer-related death among males worldwide [1]. Similar to many other malignant tumors, the clinical management of prostate cancer is still challenging due to distant metastasis, chemoresistance and relapse. Accumulating evidence shows that cancer stem cells (CSCs) are likely to play key roles in tumor relapse and metastasis, which are thought to be one of the promising targets for cancer therapy.

Warburg effect, or aerobic glycolysis, is now recognized as an important hallmark of cancers. Warburg effect, wherein glycolysis is drastically upregulated with concomitantly increased lactate acid production even in the presence of oxygen, is proven to be nearly universally prevalent in most of cancers. It was reported that in this metabolism reprogram, specific genes involved in carbohydrate metabolism in tumors lead to both upregulation of glycolysis in the cytosol and downregulation of glucose oxidation by the mitochondria [2–4]. However, the molecular mechanism behind Warburg effect in tumors is still not fully explored.

Interestingly, emerging evidence has revealed that metabolic reprograming and typical bioenergetics play vital roles in coordinating stem cell maintenance and lineage differentiation. Researches in embryonic stem cells (ESCs), hematopoietic stem cells (HSCs) and induced pluripotent stem cells (iPS) have found that all these stem cells in general have very limited oxidative capacity, but often show strong glycolysis activities. It is therefore advocated that the enriched glycolysis-dependent pathway along with reduced mitochondrial oxidative phosphorylation (OXPHOS) is a marker of cell stemness [5, 6].

Given the above findings, we hypothesized an intimate relationship between glucose metabolism switch and stemness regulation in cancer cells too. To examine the hypothesis, we chose the mitochondrial gatekeeper gene pyruvate dehydrogenase complex (*PDHc*) for further metabolic reprograming and cell stemness studies, since PDHc catalyze the bottleneck irreversible oxidation of pyruvate to acetyl-CoA thus linking the glycolytic pathway with the mitochondrial OXPHOS and its E1á subunit is the main component. By doing so, the *PDHA1* gene in the human prostate cancer cell line LnCap was knockout by TALEN technology, and the glycolysis features and cell stemness in these cells were then studied in comparison to the parental cells. We further immunohistochemically examined the expression of PDHA1 protein in a series of human prostate cancer samples and explored its relationship with pathological characteristics.

## RESULTS

### Generation of stable PDHA1 knockout LnCap cell line

**Figure 1:**
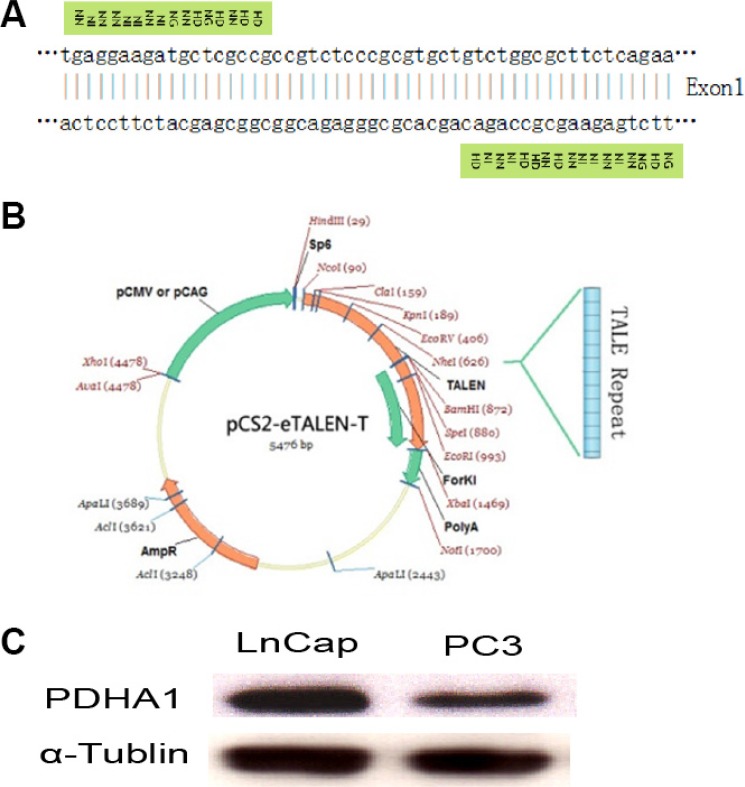
TALEN construction and cell line selection Targeting sequence of *PDHA1* gene for TALEN mediated knockout (**A**), TALEN plasmid structure (**B**) and PDHA1 expression in prostatic cancer cell lines LnCap and PC3 (**C**) are shown.

**Figure 2:**
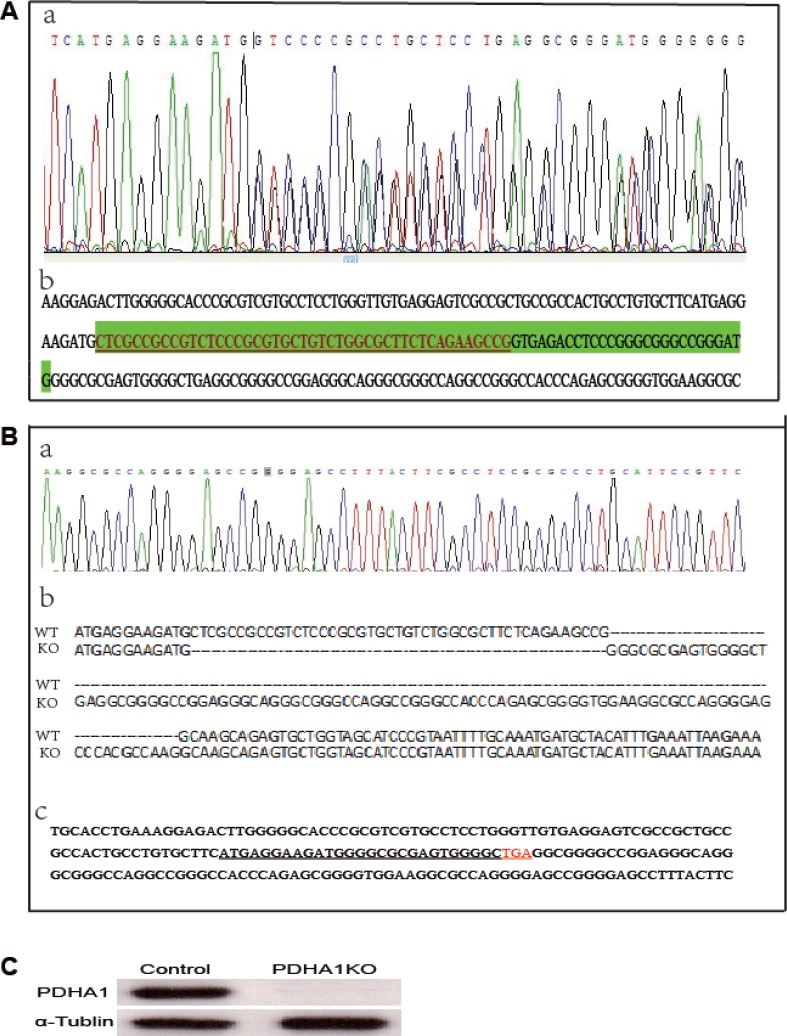
*PDHA1* gene mutation identification (**A**) The targeting area of PDHA1 extron1 was amplified from genomic DNA and the PCR fragments were sequenced to identify mutation. (a) Sequence chromatograms for the mutant type. (b) The deleted mutant bases are shown in green shade including 45 bases in the exon (with underline) and 27 bases in intron. (**B**) cDNA sequence from reverse transcriptase-polymerase chain reaction (RT-PCR). (a) Sequence chromatograms (b) Wild and mutant cDNA sequence comparison. (c) cDNA bases and the coding sequence (with underline) are shown. (**C**) The expressions of PDHA1 protein were examined by western blotting.

The TALEN repeats and target DNA sequences in PDHA1 gene are shown in Figure 1A. The TALEN-PDHA1 plasmid structure is shown in Figure 1B. To select suitable cell line for *PDHA1* gene knockout, PDHA1 protein expression was firstly examined in the prostatic cancer cell lines LnCap and PC3 by Western blotting, in order to select a cell line with higher level of PDHA1 protein expression. As shown in Figure 1C, PDHA1 protein expression was significantly higher in the LnCap cells than that in the PC3 cells by Western blotting. Therefore, to knock out *PDHA1* gene and evaluate its roles in prostate cancer cells, LnCap cells was chosen and transfected with the TALEN-PDHA1 plasmids. One stable *PDHA1* gene knockout clone (named as PDHA1KO) was established from the Lncap cell PDHA1 knockout experiments (Figure 2). The PDHA1KO cells exhibited deleted mutation in *PDHA1* gene in which 72 base were deleted in the *PDHA1* gene (Figure 2A). Figure 2B showed the mutant cDNA sequence from reverse transcriptase-polymerase chain reaction (RT-PCR).The mutation was confirmed by Western blotting, compared to the parental cells, the PDHA1 protein expression in the PDHA1KO cells was almost negative (Figure 2C). In short, the above results confirm that *PDHA1* gene was successfully knocked out in the established PDHA1KO cell line.

### Metabolic characterization of PDHA1KO cells

To gain insight into the glycolysis and OXPHOSin the PDHA1KO cells, we investigated the OCR and ECAR in the PDHA1KO cells using a Seahorse XF-24 extracellular flux analyzer. We also evaluated the glucose consumption, lactic production and ATP levels by using the corresponding kits.

**Figure 3:**
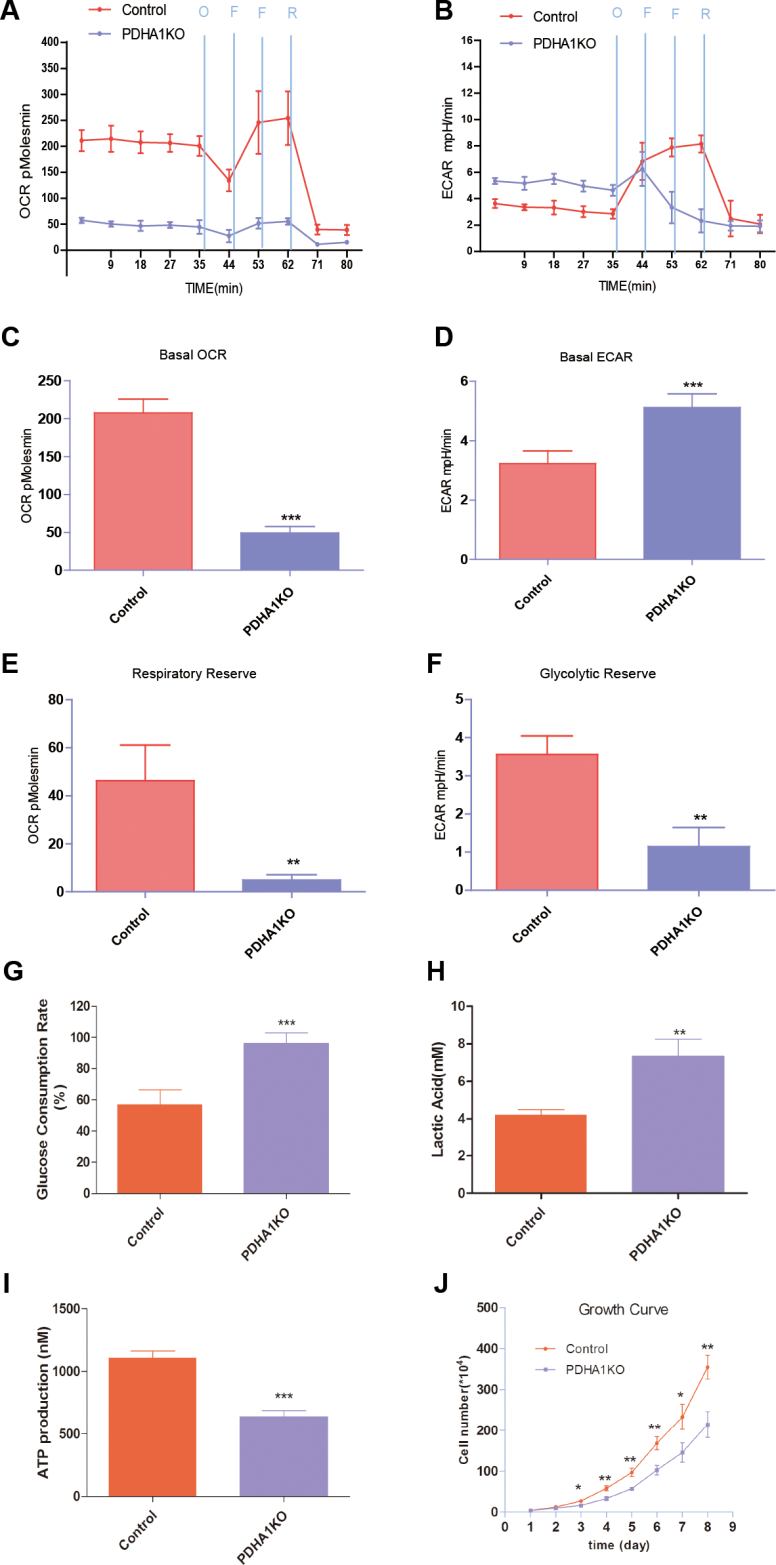
Mitochondrial respiratory profile of the cells (**A**) OCR measurements were obtained over time (min) using an extracellular flux analyzer. The mitochondrial stress test was used to obtain bioenergetics parameters, by adding Oligomycin (O, 1 mM), FCCP (F, 400 nM) (two times use of FCCP) and Reteno (R, 1 mM). (**B**) ECAR measurements were obtained as the above method. (**C**) Basal OCR. (**D**) Basal ECAR. (**E**) The respiratory reserve capacity was calculated as the difference between basal and maximal, which was stimulated by FCCP. (**F**) The glycolytic reserve capacity was calculated as the difference between basal and maximal, which was obtained when oligomycin was added. (**G**) PDHA1KO cell consumed more glucose. (H) PDHA1KO cell excreted larger amount of extracellular lactate acid. (**I**) PDHA1KO cell produced less ATP. (**J**) PDHA1KO cell showed inhibited proliferation. **p < 0.01, ***p < 0.001, Three replicated experiments were carried out with the similar results.

In the Seahorse extracellular flux analyzer system, OCR is used to measure OXPHOS and ECAR as a measurement of glycolysis. We measured OCR and ECAR under basal conditions and in the presence with oligomycin, FCCP and Reteno (Figure 3A and 3B). Basal cellular OCR of the PDHA1KO cells were found to be 49.4 ± 13.7 pmol/min per 10^6^ cells, which was significantly lower than that in the control parental cells (207 ± 33.1 pmol/min) (Figure 3C, p < 0.001) In the presence of maximally effective dose of FCCP (0.8 mM, an uncoupling agent that allows maximal electron transport), a concomitant increase of 46.3 ± 11.7 pmol/min in OCR was observed in the control cells, while there was only a slight increase of 4.9 ± 1.3 pmol/min in the PDHA1KO cells. The increase was reflected in respiratory reserve capability as shown in Figure 3E. This implies that at basal level, the PDHA1KO cells operated maximal OCR capacity, which represented a lower reserve capacity. High respiratory reserve capacity is also linked to high mitochondrial fidelity. Therefore, we were able to identify PDHA1KO cells were dysfunction mitochondrial, indicated by low basal OCR and a lack of response to FCCP.

ECAR is considered an indirect analysis of the glycolytic rate of cells. The PDHA1KO cells displayed higher basal ECAR (5.1 ± 0.8 mpH/min) than the control cells (3.2 ± 0.7 mpH/min) (Figure 3D, p < 0.01). Oligomycin was used to stimulate maximal ECAR, which shuts down ATP-dependent OCR, effectively shifting metabolism from oxidative phosphorylation to glycolysis. The difference between maximal and basal ECAR is considered the glycolytic reserve capacity of cells. According to Figure 3F, the PDHA1KO cells had lower glycolytic reserve capacity, indicating that these cells operated maximal glycolytic rate as a compensation for loss of OCR.

To further analyze the metabolic alterations, glucose consumption, lactate excretion and ATP production were evaluated. As shown in Figure 3G, the PDHA1KO cells in culture for 24 hours consumed around two folds glucose compared to the control cells. At the same time, significantly higher level of extracellular lactic acid was repeatedly revealed in the PDHA1KO cells (Figure 3H), indicating significantly more active lactate secretion of the PDHA1KO cells. To understand the impact of the *PDHA1* gene knockout on the cellular energy balance, we next analyzed ATP production in the PDHA1KO cells and the control cells. As shown in Figure 3I, ATP production was significantly lower in the PDHA1KO cells (*p* < 0.001). Collectively, the PDHA1KO cells were found unable to perform normal mitochondrial OXPHOs, but instead were forced to run anaerobic glycolysis.

### PDHA1 KO cells are slow cycling

To determine the effect of *PDHA1* gene knockout on LnCap cells proliferation, we accomplished cell growth evaluation. Growth curves demonstrated that proliferation of the PDHA1KO cells was halted compared with the control cells (Figure 3J).

### PDHA1 KO cells are chemotherapy resistant

**Figure 4:**
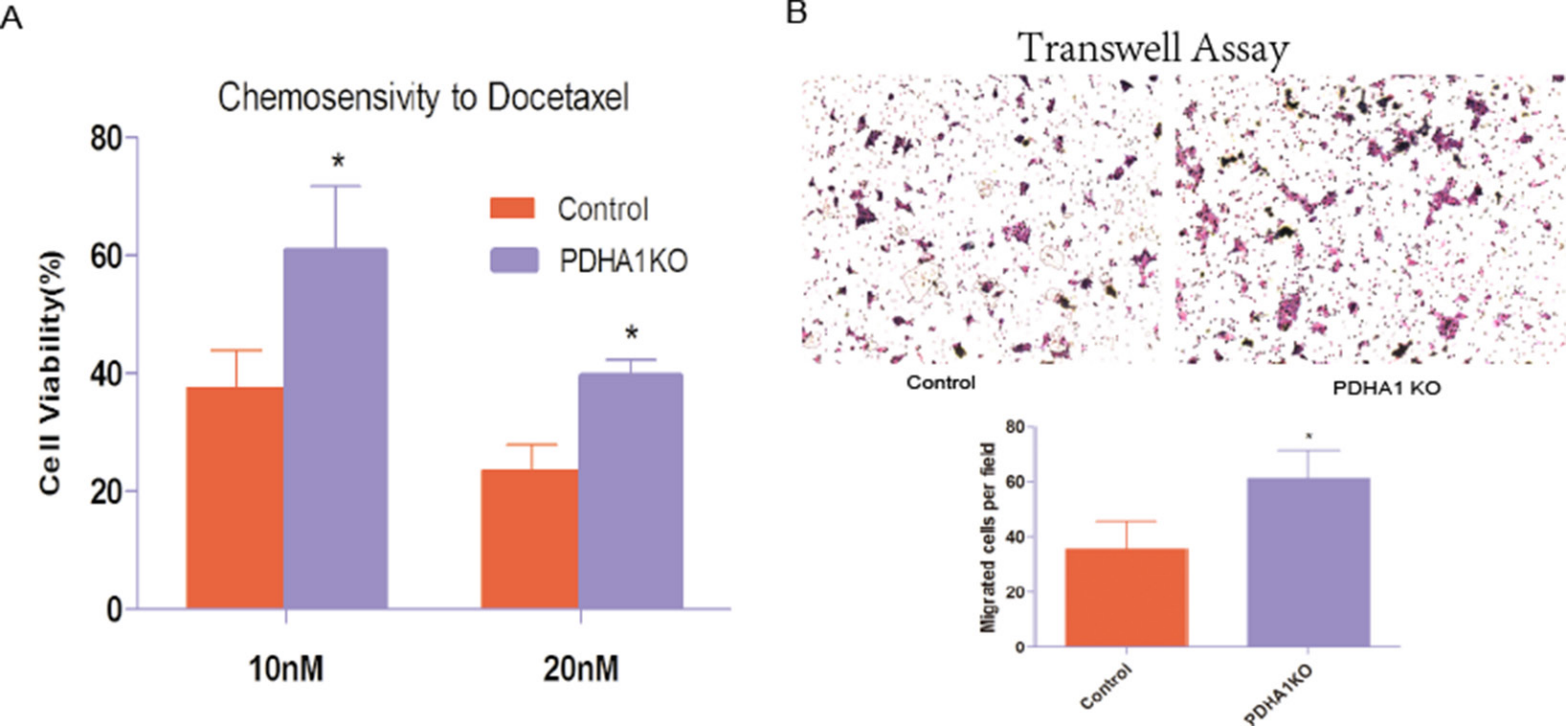
Chemotherapy sensitivity and migration ability (**A**) *PDHA1* gene knockout in the LnCap cells increased resistance to Docetaxel. All experiments were performed at least three times with consistent and repeatable results.* vs control *p* < 0.05. (**B**) Stable knockout of PDHA1 promoted migration of LnCap cell.

To evaluate whether *PDHA1* gene knockout affected the sensitivity of chemotherapeutic agents, we carried out a chemosensitivity assay. When cells were treated with escalating concentrations of docetaxel as shown in Figure 4A, the PDHA1KO cells displayed significantly better survival ability than the control cells (*p* < 0.05). These results indicate that *PDHA1* gene knockout decreases cytotoxic effects of docetaxel and enhances the chemical therapy resistance in the LnCap cells.

### The PDHA1KO cells are significantly more migratory

We further performed cell transwell assays to evaluate migration of the PDHA1KO cells and the control LnCap cells, because cell-migration ability is closely associated with the invasive growth potential. The migratory experiment results are shown in Figure 4B. The PDHA1KO cells exhibited significantly higher migration ability compared to the LnCap control cells.

### The PDHA1KO cells have higher proportion of SP cells and express higher levels of stemness markers

**Figure 5:**
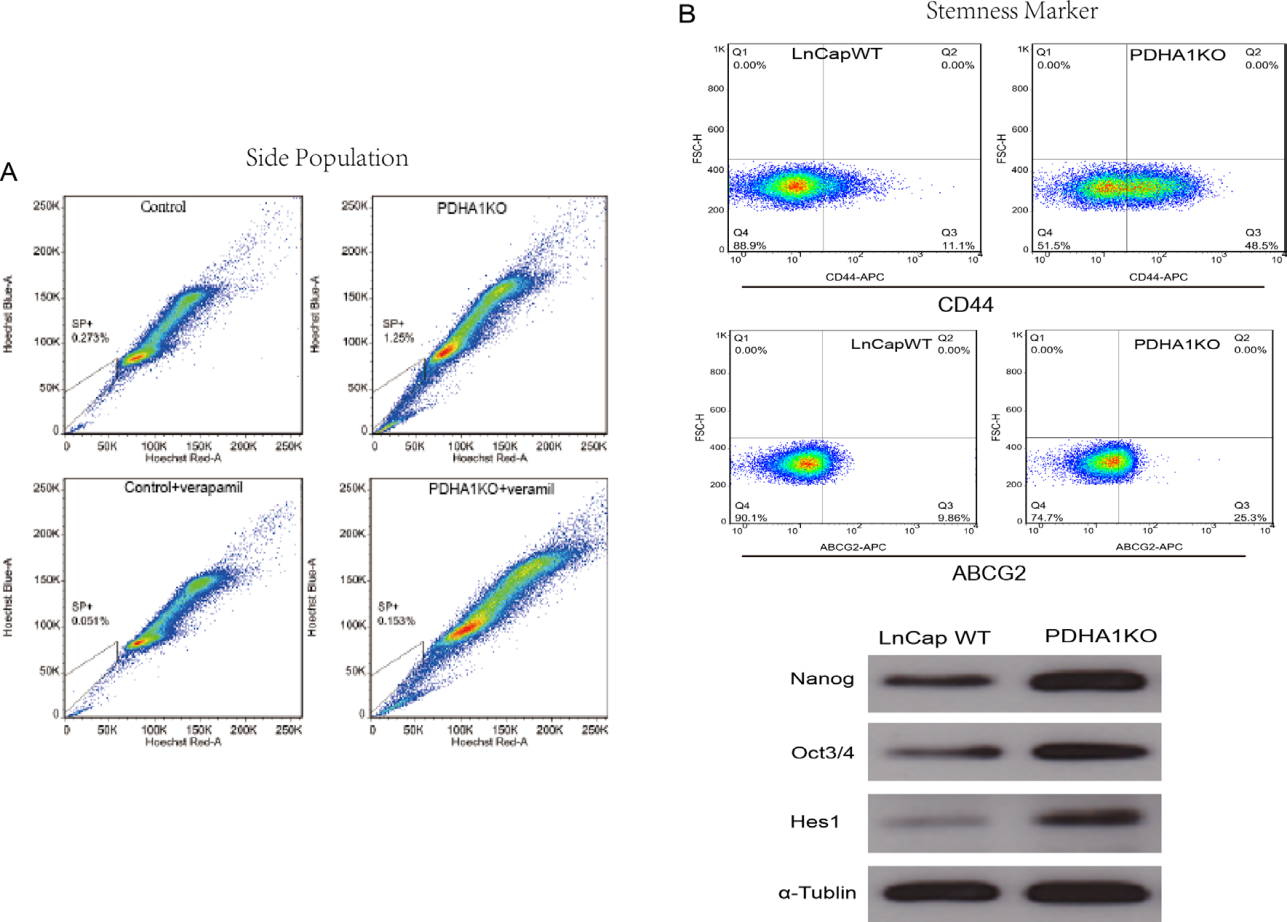
The influence of PDHA1 gene knockout on side population and the expression of stemness markers There were more side population cells in the PDHA1 KO cells,than the control cells as shown in (**A**). The expression of stem cell surface markers CD44 and ABCG2 was assessed by flow cytometry, and higher levels of CD44 and ABCG2 were identified in the PDHA1 KO cells as shown in the upper part of the B. Western blotting analyses also show higher levels of the expression of the stem cell markers Oct3/4, Nanog and Hes1 in the PDHA1 KO cells as shown in the lower part of (**B**).

We tested the proportion of the SP cells in PDHA1KO cells by using a hoechst-extrusion assay. The results revealed that the proportion of the SP was significantly increased in the PDHA1KO cell compared to the control cell (Figure 5A).

It is known that expression levels of cancer cell stemness markers are associated with cell stmness, and the higher stemness the cells show, the more stem-like the cells are. The ABCG2 and CD44 expressions in the PDHA1KO cell were examined with flow cytometry in this study. As shown in Figure 5B, significantly higher levels of ABCG2 and CD44 expressions were disclosed in the PDHA1KO cells in comparison to the LnCap control cells. Oct3/4 and Nanog play an important role in maintenance of self-renewal of embryonic stem cell and primordial germ cells. The expressions of stemness factors Oct3/4 and Nanog were investigated in this study by Western blotting. It was found that both Oct3/4 and Nanog expressions were significantly upregulated in the PDHA1KO cells. Similarly, there was significantly increasing Hes1 (hairy and enhancer of split-1) protein expression in the PDHA1KO cell as well.

### Reduced PDHA1 protein expression is correlated with poor clinicopathological characteristics

**Figure 6:**
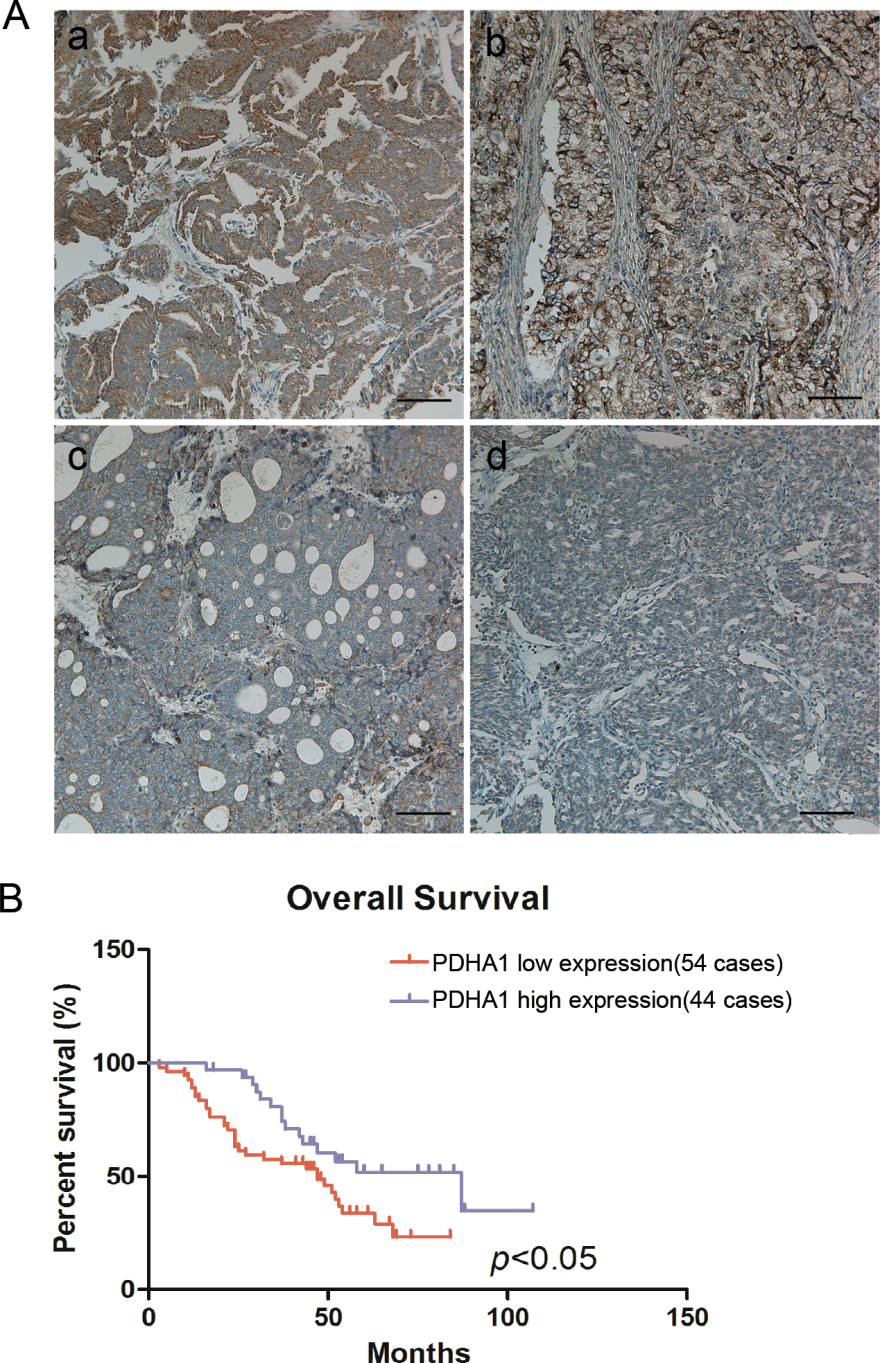
Reduced PDHA1 expression was correlated with poor prognosis Representative strong positive (a and b) and weak positive (c and d) PDHA1 expression images in prostate cancers are shown in (**A**). Kaplan-Meier survival curves show that low PDHA1 protein expression is significantly associated with worse overall survival in human prostate cancer (**B**).

To investigate whether PDHA1 protein expression abnormalities are linked to human prostate cancer in clinical samples, PDHA1 protein expression in 88 prostate cancer tissues were analyzed (Figure 6). It was discovered that 34 (38.64%) samples were strong positive, while all the other 54 tumors (61.36%) were weakly positive for PDHA1 protein expression. Representative strong positive and weakly positive immunostained slides are shown in Figure 6A. Although the expression was heterogeneous in these tumors, no single tumor was pure negative for the PDHA1 protein expression.

The associations between PDHA1 protein expression and the clinicopathological features were analyzed. As summarized in Table 1, PDHA1 expression was negativelyassociated with the Gleason score of prostate cancers. PDHA1 protein expression was noted in 15/27 (55.56%) samples with Gleason score less than 7, but only in 19/61 (31.15%) samples with Gleason score 7 or higher (*p* < 0.05).

**Table 1:** Relationship between pyruvate dehydrogenase expression and clinicopathological features of prostate cancer

Clinicopathologic variable	*n*	PDHA1 expression			
		High	Low	HR (%)	*P* value^1^
	88	34	54	62.96	
Age(year)					0.788
≤ 71	45	18	27	40.00	
> 71	43	16	27	37.21	
Gleason score					0.030
< 7	27	15	12	55.56	
7–10	61	19	42	31.15	
PSA (ng/ml)					0.088
≤ 77.56	41	11	30	26.83	
> 77.56	40	18	22	45.00	
UICC stage					0.110
pT2	67	29	38	43.28	
pT3-pT4	21	5	16	23.80	
Lymph node metastasis					0.724
Negative	74	28	46	37.84	
Positive	14	6	8	42.86	
Distant metastasis					0.515
Negative	63	23	40	36.51	
Negative	25	11	14	44.00	

^1^Pearson Chi-Square test.

Abbreviations: HR: the rate of high expression; PDHA1: pyruvate dehydrogenase; PSA: prostatic specific antigen.

OS curve was calculated by the Kaplan-Meier method and compared using the log-rank test in our study. The 3-year overall survival (OS) rate of the 88 prostate cancer patients was 66.4%, with 48 deaths observed during the follow-up period.It was demonstrated that the patients with reduced PDHA1 protein expression in the tumor tissue had worse OS than the patients with strong positive PDHA1 protein expression inthe tumor (*P* < 0.05; Figure 6B).

## DISCUSSION

We herein demonstrated that *PDHA1* gene knockout resulted in dysfunctional mitochondrial OXPHOS and enhanced glycolysis. We previously reported that impartial mitochondrial OXPHOS by using mitochondrial pyruvate carrier (MPC) blocker enhanced stemness phenotype of prostate cancer cells. In keeping with our previously study, the mitochondrial gatekeeper *PDHA1* gene knockout also leads to dysfunctional mitochondrial and enhanced glycolysis, as well as higher cell stemness phenotype. And by immunhistochemical examination of PDHA1 protein expression in prostate cancer samples, it was revealed that negative PDHA1 protein expression was related with poor clinical outcome in patients with prostate cancer.

Recent years, metabolic reprogram in tumor cells has been attracting general attention in cancer research in which tumor cells preferentially metabolize glucose through glycolysis and produce excessive lactate even in the presence of oxygen. This “aerobic glycolysis” was firstly confirmed by Otto Warburg 90 years ago and found to be a prevalent trait of cancer [8]. On the basis of this theory, positron emission tomography of 18F-2-deoxyglucose (FDG) accumulation has been used as a clinical diagnostic means of tumors. At the same time, a recent research has reported that Warburg-like metabolic change has cancer-causing activity [9]. Yi Zhu and his group identified that the nucleophosmin (NPM1) which can promote Warburg effect *in vitro* was found up-regulated in pancreatic cancers, and indicated a poor prognosis link in their study [10]. A small GTPase, which represses the Warburg effect through inhibiting GLUT1 translocation was found frequently down-regulated in lung cancers and correlated with poor prognosis as Liu J and his group reported earlier [11]. An anti-Warburg effectinitiated by fasting increased oxygen consumption was demonstrated in a short-term-starvation study [5]. Increasing evidence indicates strongly that enzymes involved in this metabolic switch process are potential targets in cancer therapy [12–15]. Arsenic, which is effective for treating some cancers, is reported to target glycolytic pathway [16]. However, the biological significance of “aerobic glycolysis” in cancer remains largely elusive.

As the main active subunit of mitochondrial “gatekeeper” PDHc, PDHA1 could be one of the most important proteins involved in the metabolism reprogram. PDHc is composed of the E1, E2 and E3 components. E1 is a heterotetramer of two á and two â subunits (20–30 copies in the complex). E1á subunit contains three conserved serine residues on which PDHC activity is regulated by two enzymes, PDH kinase (PDKs) and PDH phosphatase (PDPs), which inactivates and activates the complex, respectively [17, 18]. And in the majority of biochemically proven cases, PDHc deficiency arises from mutations in the *PDHA1* gene while rare in the genes encode E2, E3, and E3-binding protein [18]. Kavi P. Patel reviewed 371 cases of PDHc deficiency published between 1970–2010 and concluded that seventy-six percent of PDHc deficiency were due to a deficit PDHA1 [19]. Based on the above findings, we further performed *PDHA1* gene KO, and tried to better understand the role of this gene in cell metabolic reprogram and stemness regulation.

The TALEN vectors we constructed in this study, successfully deleted 72 bases in the exon 1 of *PDHA1* gene, and thus created a terminator code in the middle of exon 1, which disrupted the *PDHA1* gene translation and expression. The knockout status of *PDHA1* gene was confirmed by DNA sequening and its reduced protein expression was verified by Western blotting in the PDHA1KO cells

In the PDHA1KO cells, oxygen consumption rate (OCR) was significantly decreased, which indicated that the PDHA1KO cells consumed significantly lower amount of oxygen. The cells also concomitantly produced significantly lower level of ATP. Collectively, the *PDHA1KO* cells showed dysfunctional mitochondrial OXPHOS, Indeed, the cells were confirmed with significantly increasing ECAR, which was further verified by lactate acid assay. In parallel with these changes, the *PDHA1*KO cells consumed significantly more glucose. Based on the above findings, it is concluded that inactivation of *PDHA1* gene in the prostate cancer cells results in a metabolic reprogramming from mitochondrial OXPHOS to anaerobic glycolysis.

Cellsgenerate adenosine-5′-triphosphate (ATP) mainly through mitochondrial OXPHOS. However, the metabolic reprograming shifts from the efficient ATP production through mitochondrial OXPHOS to the significantly less effective ATP production process, glycolysis, which dramatically changes the extracellular environment in tumors due to the large amount of the excreted lactate. It has been documented that the increased lactic acid in tumors drives tumor development and progression [8, 9, 20]. Warburg even deduced that the upregulated glycolysis was caused by mitochondrial dysfunction [8], which has been challenged due to the findings that upregulated glycolysis is not always accompanied by mitochondrial defects [21]. However, increasing evidence supports a fine interplay between glycolysis and dysfunctional mitochondrial OXPHOS [22–24]. The results are largely in line with our findings, in which dysfunctional mitochondria are coupled to the Warburg effect.

Aerobic glycolysis is also the metabolic property of embryonic stem cells (ESCs), hematopoietic stem cells (HSCs) and induced pluripotent stem cells (iPSCs). In these stem cells, oxidative capacity is reduced and glycolysis is enriched. The enriched glycolysis-dependent pathway along with reduced oxidative capacity and lower mitochondrial activity has been proposed as cell stemness marker [25–27], highlighting the metabolic pathways involved in the regulation of stem cell physiology.

Cancer stem cell (CSC) is defined as a populationof cells present in tumors, which can undergo self-renewal and differentiation. It is now acceptable that CSCs exist in solid tumors with overwhelming evidence. And increasing evidence supports the key role of this portion of cells in tumor progression due to their typical roles in metastasis, and therapeutic resistance in tumors [28, 29].

Chemotherapy-resistance is considered as a trait of cancer stem cells. And docetaxel is one of the most effective cytotoxic agents in hormone resistant prostate cancer treatments. Chemosensitivity to docetaxel was therefore chosen for further analyses. It was found that the PDHA1KO cells became more resistant to docetaxel. In keeping with our result, Lyudmyla G and his group found that enforced overexpression of *PDHA1* gene caused metabolic reprograming towards mitochondrial OXPHOS and apoptosis in human liver cancer cells [30]. It is believed that distant metastasis is rooted in CSCs [31]. It is thus attemptable to investigate the cell migration ability in the PDHA1KO cells. Indeed, we found the PDHA1KO cells were significantly more migratory compared to the control parental cells, which is in line with the suggestion that mitigating the Warburg effect may specifically inhibit cancer migration [32].

We have observed that the PDHA1KO cells have the ability to expand the side population. In many cancers, these cells have been found to be more cancer stem cell-like. Stemness markers are still effective to determine cancer stem cells. CD44, ABCG2, Oct3/4, Nanog are frequently used as makers for cancer stem cells. Overwhelming evidence up to now supports CD44 could be used as a surface marker for isolating CSCs from breast, prostate, pancreas and other human cancers [33, 34]. CD44 has multifunction in cancer cells, like acts as a specific receptor for promoting migration, interacts with osteopontin and regulates its cellular functions leading to tumor progression, monitors the extracellular changes and is vital in regulating cell survival, migration and differentiation [33, 35, 36] [37, 38]. Hes1, also considered as stem cell marker, is able to promote migration and invasion of nasopharyngeal carcinoma cells [39]. As expected, the PDHA1KO cells expressed significantly higher levels of CD44, ABCG2, Oct3/4, Nanog and Hes1. All these results show that loss of PDHA1 gene expression significantly upregulates cell stemmness of prostatecancer cells *in vitro*, which strongly indicates its negative role in the development of prostate cancers.

In human prostate cancer tissues, we showed that reduced PDHA1 protein expression predicted worse clinical outcome. Using an immunochemical staining approach, we found about 61.4% (54 of 88 samples) of prostatic cancer samples with reduced PDHA1 protein expression. Interestingly, PDHA1 was shown to associate with the grades of tumor differentiation. In the overall survival analysis, reduced PDHA1 staining was associated with poor overall survival. In short, reduced PDHA1 protein expression predicted worse clinical outcome in prostatic cancer.

To date, we are the first to knock out PDHA1 gene and successfully establish a PDHA1KO prostate cancer cell line with TALEN technology. Compared to the parental control cells, the PDHA1KO cells consume significantly less amount of oxygen and produce higher amount of extracellular lactate acid. The cells consume significantly more glucose and synthesize significantly less ATP. All the above results confirm that the PDHA1KO cells show metabolic reprogramming towards anaerobic glycolysis. Furthermore, the PDHA1KO cells express higher levels of cancer cell stemness factors, and these cells are migratory and resistant to chemotherapy *in vitro*. Clinically, reduced PDHA1protein expression in prostate cancers is associated with aggressive features and poor overall survival in our currently study. Collectively, the above results indicate that the function of PDHA1 is associated with the metabolic reprogramming and the cell stemness in prostate cancer cells, negative protein expression of this gene is associated with poor clinical outcome of prostate cancers.

## MATERIALS AND METHODS

### Cell lines and cell culture

Prostatic cancer cell lines LnCap and PC3 were obtained from ATCC (American Type culture collection, USA) and maintained in our laboratory for the study. The cells were cultured in RPMI 1640 medium Gibco-BRL) supplemented with 10% fetal bovine serum (FBS, Gibco), 100 U/ml of penicillin and 100 ug/ml streptomycin at 37°C, and 5% CO2.

### TALEN construction and activities evaluation

Using TALEN design software (http://tale-nt.cac.cornell.edu/), one 16-mer repeat variable di-residue (RVD) TALEN and one 17-mer RVD TALEN with a 17-bp spacer were designed to target exon 1 of the human *PDHA1* locus.

To assess the endonuclease-dependent genome editing activities, 72 hours after transfection gDNA was prepared from the transfected cells.The targeted region on *PDHA1* gene was amplified by PCR followed by being digested using T7E1 enzyme (BioLabs M0320). The primer sequences flanking the targeted region are 5′-GATACCCAATGGGCAGCCTC-3′ and 5′- AGAAGGGGGAAGTTCACACG-3′.

### Transfection

For transfection, the cells were seeded in six-well plates and allowed to grow overnight to 60%–70% confluent. The cells were then transfected with a mixture of 2 μg TALEN pair plasmid and 10 μl lipofectamine 2000 (Invitrogen) in 2 ml serum-free medium. At 24 hours after transfection, the medium was replaced by normal medium containing 10% FBS.

### Generating stable *PDHA1* gene knockout cell line

Seventy-two hours after transfected with the TALEN-PDHA1 plasmid the cells were harvested to produce single cell suspension by using limiting dilution preparation and single cell was placed in 96-well plate for monoclonal cell growth. Genome DNA of the monoclonal cells was prepared afterwards. The targeted region of *PDHA1* gene was amplified by the above PCR primers and all the PCR products were subjected to sequencing in the cell line establishment period. The clones confirmed to be mutated were subjected to further protein level confirmation.

### DNA preparation and PCR

DNA was extracted from approximately 10^5^ cells using Genomic DNA Mini Kit (Invitrogen). The primers were: forward 5′-GATACCCAATGGGCAGCCTC-3′ and reverse 5′-AGAAGGGGGAAGTTCACACG-3′, with product length of 574 bp. 1 ng gDNA was added to the PCR system under the following PCR program: initial denaturation at 98°C for 2 minutes; followed by 30 cycles of 98°C for 10 seconds, 68°C for 30 seconds and 72°C for 30 seconds; then 68°C for 10 minutes.

### RNA preparation and RT-PCR

RNA was extracted from approximately 10^5^ cells using Rneasy Micro kit (Qiagen). The RNA was used as template to synthesize cDNA as follows. RNA(1 ug) was added to the reverse transcription system under the following program: 37°C for 10 minutes and 42°C for 60 min, followed by 72°C for 40 min; 4°C for 4min. After that the cDNA was go for sequencing.

### Western blotting

Cells subjected for Western blotting were homogenized using RIPA buffer (Thermo Scientific Pierce Company). Then the homogenates were centrifuged and the supernatants were collected for immunoblotting analysis. Briefly, equal amount of 30 μg of cellular proteins were loaded onto a 10% SDS-PAGE. After electrophoresis, the proteins were transferred to polyvinylidene difluoride transfer membrane (PVDF) in a trans-blot apparatus (Bio-Rad,Hercules,CA). Membranes were blocked in 5% milk, and incubated with the indicated antibodies. After washing with PBST (PBS with 0.1% Tween) corresponding secondary antibodies conjugated with horseradish peroxidase-conjugated (HRP) were then added. Finally, the membranes was washed and detected by enhanced chemiluminescence (Amersham, Arlington Heights, IL) and exposed to X-ray film.

### Measurement of oxygen consumption rate (OCR) and extracellular acidification rate (ECAR)

OCR and ECAR were measured using a Seahorse XF24 extracellular flux analyzer (Seahorse Bioscience, North Billerica, MA, USA). Briefly, cells were plated at 1 × 10^5^ cells/well in a XF24 cell culture microplate (Seahorse Bioscience) and cultured for 6 hours to attach to the bottom. The cells were switched into unbuffered DMEM supplemented with 2 mM sodium pyruvate prior to the beginning of the assay and maintained at 37°C. OCR was reported in the unit of picomoles per minute and ECAR was reported in milli-pH units (mpH) per minute. After baseline measurements, OCR and ECAR were measured after sequentially adding to each well 20 μl of oligomycin, FCCP and rotenone, to reach working concentrations of 1 μg/ml, 0.4 μM and 1 μM, respectively.

### Determination of glucose consumption, lactate and ATP production

1 × 10^6^ cells per well were seeded and cultured for 24 hours and the cell culture medium was tested for glucose consumption and lactate assay by using a glucose assay kit (GAHK-20, Sigma) and EnzyChrome lactate assay kit (ECLC-100, BioAssay), respectively, according to the manufacturer's instructions. For cellular ATP production determination, 1 × 10^6^ cells were harvested and cell lysis was achieved by sonication (in ice-water bath) before cellular ATP was detected with a Molecular Probes’ ATP Determination Kit (Life technology) according to the manufacturer's instructions.

### *In vitro* cell growth assay

The cells were seeded at 20000 cells per well in 6-well plates (NUNC^TM^, Thermo Scientific, Denmark). After culturing for 1, 2, 3, 4, 5, 6, 7 and 8 d, cells were brought into suspension using trypsin. Trypan blue was added to the cell suspension and unstained cells were counted.

### Chemosensitivity assay

Cells were seeded in 25 cm^2^ flasks at a density of 1 × 10^6^ cells per flask. Docetaxel (10, 20 nmol/L) were added to different flasks 12 hours after seeding. After 72 hours, cells were brought into suspension and trypan blue was added to the cell suspension for counting unstained cells. Cell viability was calculated based on the ratio of unattained and stained cells. The experiments were conducted in triplicate.

### Transwell cell migration assay

Cell migration was examined using transwell filters with 8 um pore (Corning, USA). Cells (4 × 10^4^) in 200ul of serum-free medium were added to the upper chamber, and the lower chambers were filled with 750 ul complete medium. The cells were allowed to migrate at 37°C in a 5% CO2 humidified incubator for 16 hours. Non-migrating cells on the upper surface were carefully removed with a cotton swab. The filters were then fixed in ethanol for 10 minutes and stained with crystal violent for 20 minutes. Migrated cells on the membrane were counted under a microscope. Migration was quantified by counting the migrated cells in 10 random high-powered fields per filter.

### Flow cytometry

After harvesting cells with 0.05% trypsin-EDTA solution, single cell suspensions were washed and blocked using 5% Rabbit serum (Invitrogen 705784A) in a permeabilisation buffer (PBS with 0.5% TritonX100) for 1h at 37°C and stained with appropriate CD44 and ABCG2 antibodies conjugated with fluorophores at 37°C avoid from light for 25 min. For each sample, an unstained sample and isotype control antibody stained sample were prepared for gating. Cell suspensions were washed twice with PBS and resuspended in HBSS+ buffer. Before test, 1 μl 1mg/ml PI was added to exclude dead cells. Samples were analyzed on a BD LSRII flowcytometer. Software Flowjo version 7.6 was applied for data analyze and present.

### Clinical samples

In total, 88 surgically dissected prostate cancer samples were included in this study.All patients underwent transurethral electrovaporization of prostate or radical prostatectomy from Dec 2005 to Dec 2011 at the First Affiliated Hospital of Zhengzhou University. The average age is 71 years (55–92 years). The detailed clinicopathological features are summarized in Table 1. All patients were followed up from the confirmed date of diagnosis until death or 31 March 2015. Two pathologists at the Department of Pathology of the First Affiliated Hospital of Zhengzhou University reviewed the type and grade of histology of the specimens.Ethical approval for this research was obtained from the Research EthicsCommittee of Zhengzhou University, China. All the patients involved provided written informed consent.

### Immunohistochemistry and staining evaluation

The formalin-fixed, paraffin-embedded sections were obtainedfrom the Department of Pathology,the First Affiliated Hospital of Zhengzhou University. Dako EnVisionTM Flex+ (K8012; Dako, Glostrup, Denmark) was applied for IHC staining as the IHC mentioned. Negative controls were performed using the same concentration of non-immune rabbit IgG instead of the rabbit anti-human PDHE1á subunit antibody. Tumor cell positivity was scored according to both intensity and extent of staining [17]. The intensity was scored as follows: 0, no positive cells; 1, weak staining; 2, moderate staining; and 3, strong staining. The extent of staining was scored according to the percentage of immunostained cells as follows: 0, no positive cells; 1: < 1%; 2: 1–10%; and; 3:11–33%; 4: 34–66%; 5: 67–100%. The sum of intensity score and extent score was taken as total score, which ranged from 0 to 8. The slide was regarded as weakly positive for PDHA1 protein expression when the total score was ≤ 3, and the slide was regarded as strong positive when the total score was ≥ 4.

### Antibodies

Antibodies against human PDHA1(cell signaling, C54G1), tubulin(sigma,103M4773), CD44(BD pharmingen 559942), ABCG2(BD pharmingen,561451), Nanog(cell signaling 1E6C4), Sox2(RD, MAB2018) and Oct3/4(RD, AF1759 in addition to the APC isotype control( BD pharmingen 555745) and the second antibody goat anti-mouse(Life technology, 1484573) were applied in this study.

### Statistical analyses

Statistical analyses were performed using SPSS 17.0 software (SPSS Inc, Chicago, IL, United States). All data of the *in vitro* analyses were obtained from at least three independent experiments and were analyzed by the one-way ANOVA test and student's *t* test. Overall survival (OS) rates of the patients were determined using Kaplan-Meier curves. The log-rank test was used to identify the differences between survival curves. Chi-square tests (Pearson as appropriate) were performed foranalyzing the associations of PDHA1 expression and clinicopathological variables. *P* < 0.05 were considered significant.
